# Crystal Structure of *Escherichia coli*-Expressed *Haloarcula marismortui* Bacteriorhodopsin I in the Trimeric Form

**DOI:** 10.1371/journal.pone.0112873

**Published:** 2014-12-05

**Authors:** Vitaly Shevchenko, Ivan Gushchin, Vitaly Polovinkin, Ekaterina Round, Valentin Borshchevskiy, Petr Utrobin, Alexander Popov, Taras Balandin, Georg Büldt, Valentin Gordeliy

**Affiliations:** 1 Institute of Complex Systems (ICS-6) Structural Biochemistry, Research Centre Jülich GmbH, Jülich, Germany; 2 Laboratory for advanced studies of membrane proteins, Moscow institute of physics and technology, Dolgoprudniy, Russia; 3 Univ. Grenoble Alpes, IBS, Grenoble, France; 4 CNRS, IBS, Grenoble, France; 5 CEA, IBS, Grenoble, France; 6 European Synchrotron Radiation Facility, Grenoble, France; University of Saskatchewan, Canada

## Abstract

Bacteriorhodopsins are a large family of seven-helical transmembrane proteins that function as light-driven proton pumps. Here, we present the crystal structure of a new member of the family, *Haloarcula marismortui* bacteriorhodopsin I (*Hm*BRI) D94N mutant, at the resolution of 2.5 Å. While the *Hm*BRI retinal-binding pocket and proton donor site are similar to those of other archaeal proton pumps, its proton release region is extended and contains additional water molecules. The protein's fold is reinforced by three novel inter-helical hydrogen bonds, two of which result from double substitutions relative to *Halobacterium salinarum* bacteriorhodopsin and other similar proteins. Despite the expression in *Escherichia coli* and consequent absence of native lipids, the protein assembles as a trimer in crystals. The unique extended loop between the helices D and E of *Hm*BRI makes contacts with the adjacent protomer and appears to stabilize the interface. Many lipidic hydrophobic tail groups are discernible in the membrane region, and their positions are similar to those of archaeal isoprenoid lipids in the crystals of other proton pumps, isolated from native or native-like sources. All these features might explain the *Hm*BRI properties and establish the protein as a novel model for the microbial rhodopsin proton pumping studies.

## Introduction

Microbial rhodopsins are a large family of seven-helical transmembrane proteins that contain the covalently attached cofactor retinal [1]. Upon absorption of a photon, the retinal isomerizes and starts a series of structural transformations, correlated with spectral changes and called photocycle [1], [2]. Among the members of the family are light-driven proton, anion or cation pumps, light-gated ion channels and photoreceptors [1].

The most studied class of microbial rhodopsins are the proton pumps. It has been established that there are three distinct regions involved in the proton translocation: the proton donor site, the retinal binding pocket, and finally, the proton release region. In *Halobacterium salinarum* bacteriorhodopsin (*Hs*BR), identified in 1971 by Oesterhelt and Stoeckenius [3], the proton translocation cycle starts with isomerization of the retinal and transfer of the proton from the retinal Schiff base to the side-chain of the proton acceptor D85 [2]. In the next stage, the proton is transferred to the proton release group, consisting of two closely situated glutamates E194 and E204 and several water molecules. Then, the Schiff base is reprotonated from the proton donor D96, which itself is reprotonated from the cytoplasm. Although the general details of this proton pumping mechanism are well known, there are some discrepancies between the published structures of the intermediate states [4], [5], some of which might be explained by high radiation susceptibility of bacteriorhodopsin [6] or twinning of crystals [7]. Consequently, different details of the bacteriorhodopsin proton pumping still continue to be investigated, related to both the proton release [8] and the proton uptake [9].

In the recent years, structures of many microbial rhodopsins have been determined. Structures of four different archaeal proton pumps are known, *Hs*BR [10], [11], archaerhodopsin-1 and -2 (ar-1 and ar-2) [12], and deltarhodopsin-3 (dr-3) [13], as well as the structures of bacterial pumps xanthorhodopsin [14], various proteorhodopsins [15], [16] and eukaryotic *Acetabularia* rhodopsin [17] and *Chlamydomonas reinhardtii* channelrhodopsin [18]. Here, we present the structure of bacteriorhodopsin I from *Haloarcula marismortui* (henceforth *Hm*BRI). *Hm*BRI is one of the six *Haloarcula marismortui* retinylidene proteins and one of its two bacteriorhodopsins, whose sequences are 50% identical [19]. The protein can be expressed in *Escherichia coli* in large quantities and in fact can be used as a tag for production of other membrane proteins [20]. The structure reveals the conserved proton donor and retinal-binding pocket sites, and expanded proton release region. There are three additional inter-helical hydrogen bonds in the *Hm*BRI transmembrane region. Despite heterologous expression, the protein assembles as a trimer in crystals. The loop between the helices D and E of *Hm*BRI is extended, makes contacts with the adjacent protomer and appears to stabilize the interface. These features might result in a higher stability of *Hm*BRI and explain its high expression level in *E. coli* cells. *E. coli* production allows for easy genetic manipulation and rapid production of mutants, which will be helpful for studies of the proton transport mechanism as well as for protein engineering for optogenetics needs [1], [21].

## Results and Discussion

### HmBRI expression and spectroscopic characterization

**Figure 1:**
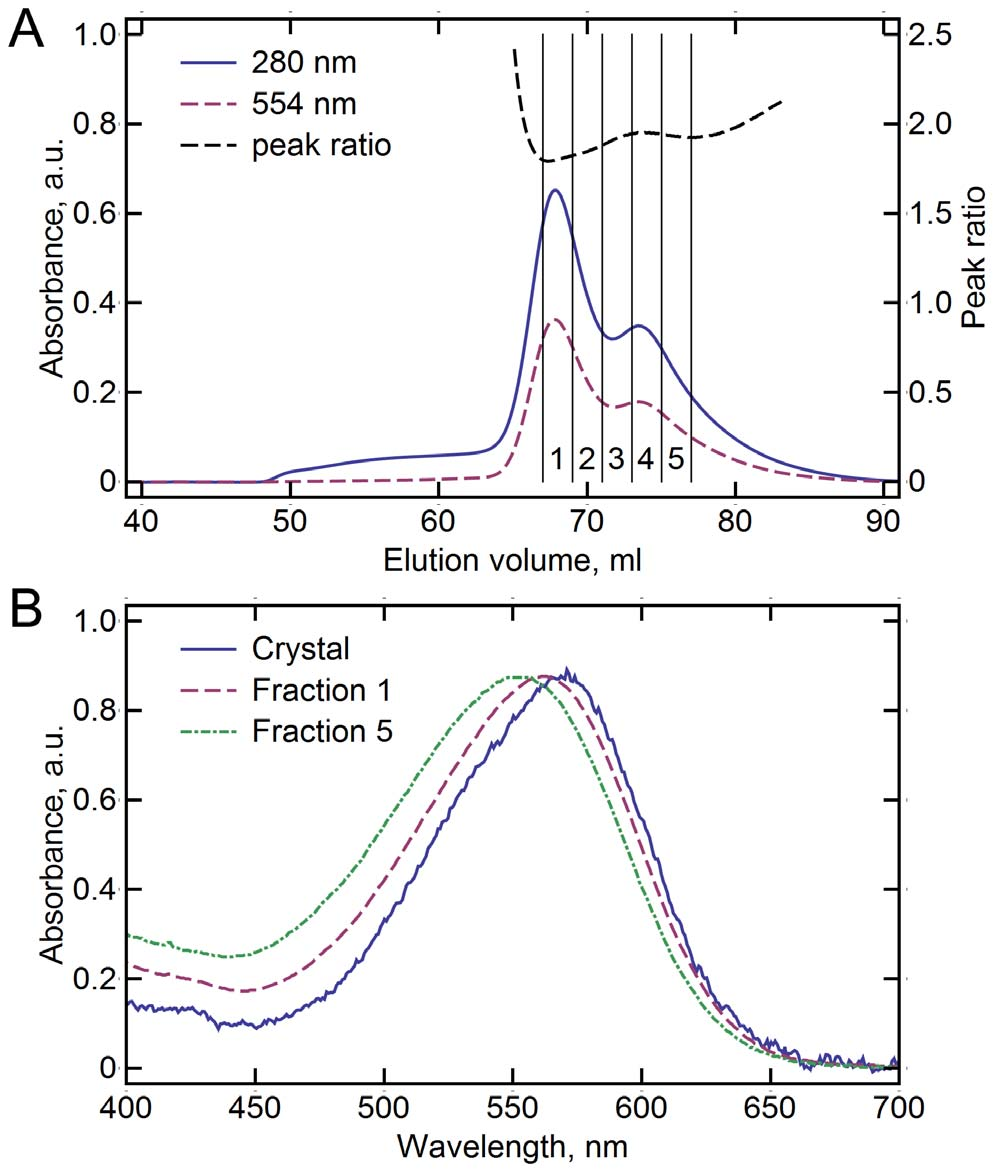
*Hm*BRI size-exclusion chromatography (SEC) elution profile and visual light absorbance spectra in different environments. (A) SEC elution profile of *Hm*BRI. Two distinct peaks are observed, that correspond supposedly to monomeric *Hm*BRI (at 73.4 ml, peak ratio is ∼1.95) and oligomeric *Hm*BRI (at 67.8 ml, peak ratio is ∼1.85). Five 2 ml fractions (1–5) were collected for subsequent crystallization. (B) Spectra of the light-adapted *Hm*BRI in the hexagonal crystals, the first and the fifth SEC fractions.

*Hm*BRI was heterologously expressed in *E. coli* and purified by nickel-affinity and size-exclusion (SEC) chromatography. The SEC elution profile revealed that the protein exists in two forms in the detergent solution (Figure 1A), presumably monomeric and oligomeric (most probably trimeric). Both forms behave identically on the sodium dodecyl sulfate polyacrylamide electrophoresis. Although the approximate molecular weights of the two species can be calculated as 130 kDa (the lighter form) and 210 kDa (the heavier form) on the basis of their elution volumes, this information is not enough to determine the oligomeric states, as the relation between the SEC mobility of a protein and its molecular weight is not accurate. Also, the size of the detergent micelle around the protein and amount of the lipids of the expression host carried with the protein are not known.

To determine the potential differences between the two forms, the protein was characterized spectroscopically. The lighter form has the absorption maxima at 554 nm in the light-adapted state, and the heavier form at 562 nm (Table 1). The absorption maxima of both forms are blue-shifted by ∼4 nm in the dark-adapted state. Thus, the protein's absorption maximum depends on its oligomeric state. The small variation of the absorbance maximum position with dark adaptation as compared to other bacteriorhodopsin-like proton pumps might be explained by the nature of the retinal β-ionone ring-proximal side-chain, corresponding to M145 in *Hs*BR. It has been shown, that the M145F *Hs*BR mutation, reducing the steric conflict, results in much lower occupancy of the 13-*cis* retinal conformer in the dark-adapted state and a smaller absorption maximum shift [22]. In *Hm*BRI, the residue is L149, and its position is closer to that of F145 in the ar-2 structure, than to that of M145 in *Hs*BR or ar-1 structures [12], [23].

**Table 1:** *Hm*BRI absorption maxima in different environments (nm).

Environment	Dark-adapted state	Light-adapted state
Crystal	n/d	∼566
*H. salinarum* polar lipids	559	563
DDM Fraction 1* (SEC)	559	562
DDM Fraction 3* (SEC)	552	555
DDM Fraction 5* (SEC)	549	554

* See Figure 1A for definition of the SEC fractions.

*Hm*BRI, reconstituted into *H. salinarum* polar lipids, revealed the spectroscopical characteristics very similar to those of the heavier detergent form. Such red shift of the absorption maximum of the protein in the membrane relatively to the absorption maximum in the detergent solution was also observed in the case of *Hs*BR [24], [25]. Finally, *Hm*BRI in crystals, where it is expected to be in the light-adapted state, absorbed at even higher wavelengths (Figure 1B, Table 1).

Since *Hm*BRI, similarly to others light-driven archaeal proton pumps, is expected to form trimers in the native membrane (and forms trimers in crystal), and the heavier detergent form absorbs light similarly to the protein reconstituted into the native-like membranes, we suggest that the two observed forms of the protein in detergent are monomers and trimers.

### Crystallization and crystal packing of HmBRI

**Figure 2:**
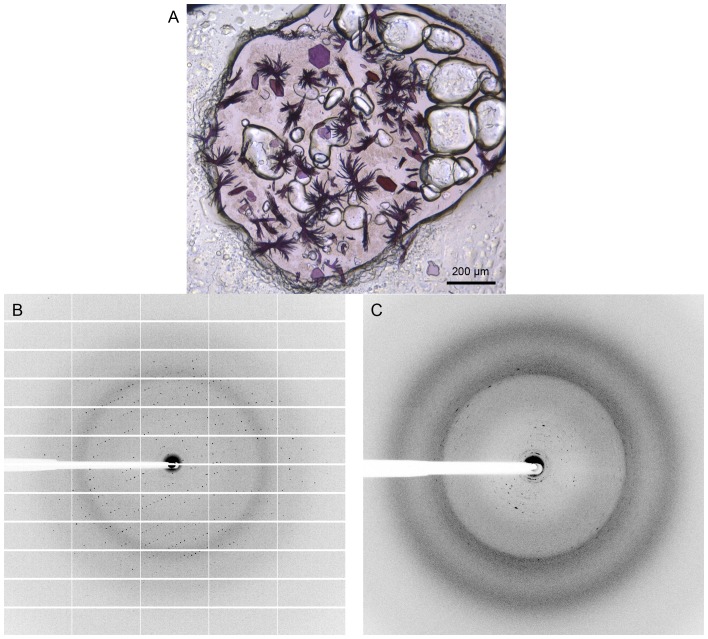
*Hm*BRI crystals and diffraction. (A) *Hm*BRI crystals in the crystallization well. Two types of crystals are observed: regular shaped hexagonal crystals and bunches of needles. (B) Example of the diffraction from the hexagonal crystals. Resolution at the detector edge is 2.4 Å. (C) Example of the diffraction from needle-shaped crystals. Resolution at the detector edge is 2.5 Å. The diffraction patterns are smeared and observed up to the resolution of 3.5 Å.

The *Hm*BRI protein was crystallized using the *in meso* approach [26], [27] similarly to our previous work [15], [28]. Two types of crystals appeared in the same crystallization wells: regularly shaped hexagonal crystals and bunches of needles (Figure 2A), both approaching 100 µm in the largest dimension. Absorption maximum of *Hm*BRI in the hexagonal crystals is at ∼567 nm (Figure 1B). While the hexagonally shaped crystals produced good diffraction patterns up to the resolution of 2.3 Å (Figure 2B, Table 2), the needles produced overlapping diffraction patterns up to the resolution of 3.5 Å, which could not be indexed (Figure 2C).

**Table 2:** Crystallographic data collection and refinement statistics.

Data collection	
Space group	P321
Cell dimensions	
*a*, *b*, *c* (Å)	102.85, 102.85, 60.00
α, β, γ (°)	90, 90, 120
Resolution (Å)	60–2.5 (2.63–2.5)*
*R*_merge_ (%)	15.4 (94.9)*
*I*/*σI*	9.1 (3.0)*
*CC1/2*	0.99 (0.64)*
Completeness (%)	98.7 (97.5)*
Multiplicity	5.4 (5.4)*
Refinement	
Resolution	60–2.5 Å
No. reflections	12817 (623**)
*R*_work_/*R*_free_	19.16%/23.72%
Number of atoms	
Protein and retinal	1827
Water	19
Lipid fragments	188
Sulfate ions	10
Average B-factor (Å^2^)	
Protein and retinal	38.8
Water	40.8
Lipid fragments	53.5
Sulfate ions	63.9
R.m.s. deviations	
Bond lengths	0.010 Å
Bond angles	1.7°

* Values in parentheses are for the highest-resolution shell.

** Number of reflections that are not used for refinement (free reflections).

**Figure 3:**
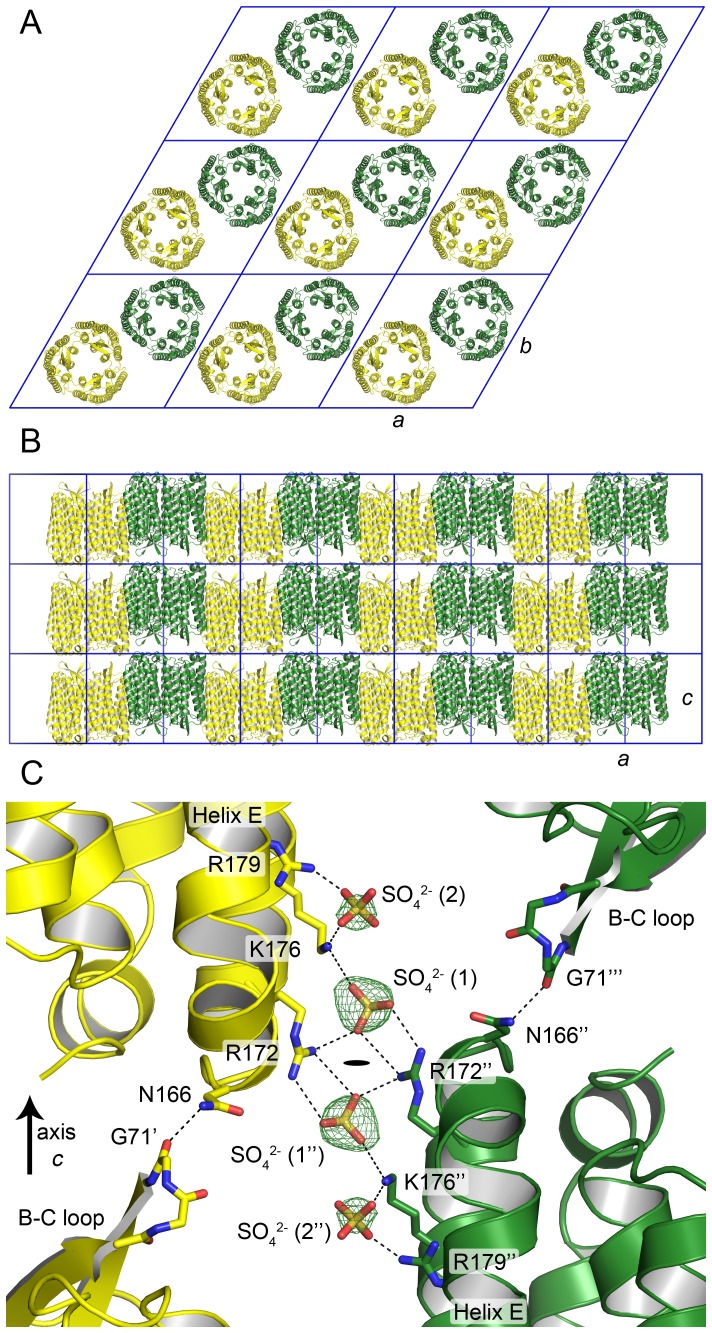
*Hm*BRI packing in hexagonal crystals. (A) Packing in the membrane plane. There are two trimers in the unit cell (shown in yellow and green) that are oriented in opposite directions. (B) Packing of the membrane-like layers. (C) Crystal contacts between the layers. The contacts rely on the ionic interactions between the SO_4_^2−^ ions and the R172 and K176 side-chains, and on the hydrogen bond between the N166 side-chain of one protomer and the G71 backbone oxygen of another. A second putative SO_4_^2−^ ion is observed close to the first one that interacts with K176 and R179 side-chains. Potential interactions are marked by dashed lines. F_o_-F_c_ difference electron densities before the insertion of the ions into the model are shown at the level of 3 σ. The symbols ', ” and ’” denote different crystallographic symmetry-related HmBRI molecules. The black oval denotes the crystallographic symmetry rotation axis (C_2_).

The spacegroup of the hexagonally shaped crystals was determined to be P321, cell parameters *a*, *b* = 102.9 Å, *c* = 60.0 Å. The protein is in the same arrangement as archaerhodopsin-2 in the trimeric form (PDB ID 2EI4 [29], space group P321, cell parameters *a*, *b* = 98.2 Å, *c* = 56.2 Å). Typically for *in meso* crystallization, *Hm*BRI crystals are organized as stacks of the membrane-like layers (Figure 3A). The unit cell spans one such layer, and contains two *Hm*BRI trimers, oriented in opposite directions (Figures 3A and 3B).

The inter-layer crystal contacts rely on the ionic interactions between the sulfate ions and the side-chains of the helix F lysine 176 and arginine 172 of the opposing *Hm*BRI molecules (Figure 3C). The second partially occupied sulfate ion binding site is observed close to the first one. However, the second sulfate does not participate in inter-molecular contacts and is bound to the residues K176 and R179 from the same *Hm*BRI molecule. A sulfate ion at the similar position is observed in the structure of deltarhodopsin-3 [13], where it is bound by R160 of the helix E and R174 of the helix F. The other inter-layer contact in *Hm*BRI crystals is formed by the hydrogen bond between the G71 oxygen of one *Hm*BRI molecule and N166 amine nitrogen of another (Figure 3C).

The intra-layer contacts between the *Hm*BRI trimers are mediated mostly by the ordered hydrophobic tail groups of the lipid molecules, and by the two symmetric hydrogen bonds between the amine nitrogen of N180 from the helix F of one protomer and the backbone carboxyl of L202 from the F-G loop of another.

### Structure of HmBRI

**Figure 4:**
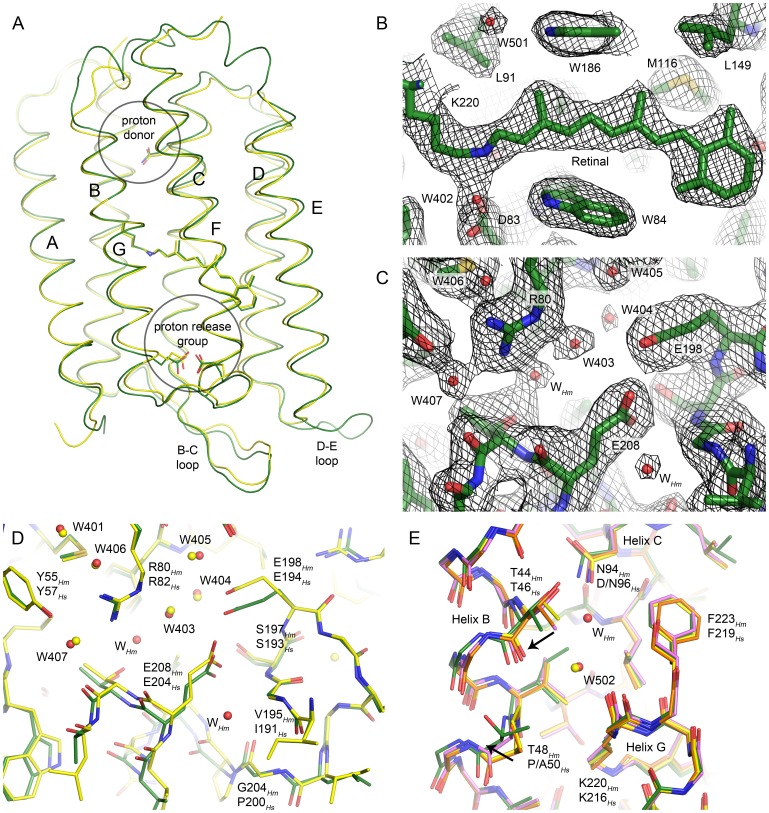
Crystallographic structure of the *Hm*BRI D94N mutant. (A) Comparison of the *Hm*BRI backbone structure (green) with that of *Hs*BR [23] (yellow). (B) 2F_o_-F_c_ electron density maps in the vicinity of the retinal. The maps are contoured at the level of 1.5 σ. (C) 2F_o_-F_c_ electron density maps in the proton release region. The maps are contoured at the level of 1.2 σ. (D) Comparison of the *Hm*BRI proton release group (green) with that of *Hs*BR [23] (yellow). Overall, the conformations of the side-chains and positions of water molecules are very similar. However, the water accessible space is larger in *Hm*BRI, and additional water molecules are observed (W*_Hm_*). One of the reasons for this difference might be the substitution of *Hs*BR’ proline 200 with the glycine 204 in *Hm*BRI, that allows unlatching of the extracellular part of the helix G. (E) Comparison of the *Hm*BRI proton donor region with that of wild-type *Hs*BR [23] (green) and its D96N [30] (orange) and P50A [32] (magenta) mutants. It appears that in *Hm*BRI the effects of the D94N mutation and the P50_Hs_ → T48_Hm_ substitution combine and result in a larger displacement of the helix B relative to the helices C and G (black arrows), as similar displacements are present in the P50A and D96N mutants of HsBR, albeit with a smaller amplitude.

**Figure 5:**
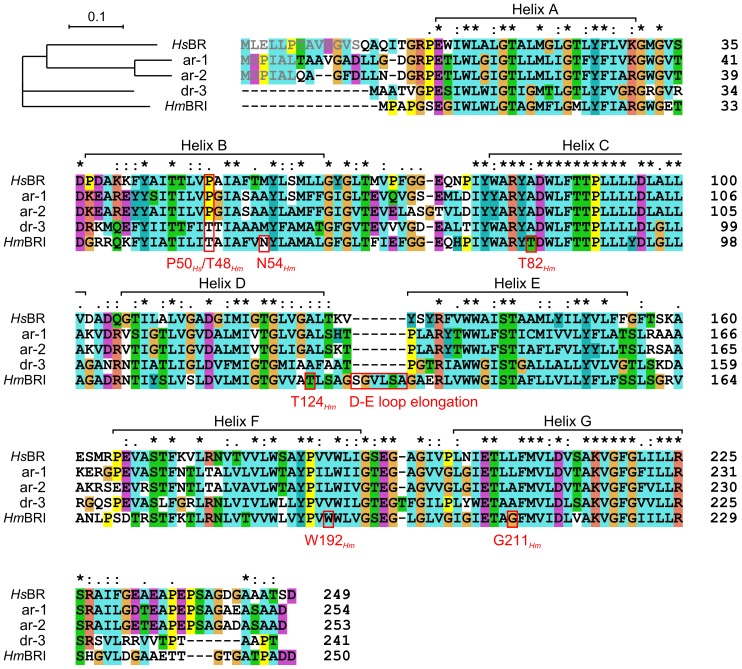
Phylogenetic tree and sequence alignment of *Hs*BR, ar-1, ar-2, dr-3 and *Hm*BRI. The unique *Hm*BRI D-E loop elongation, the residues that result in novel inter-helical hydrogen bonds, and the P50*_Hs_*/T48*_Hm_* position are highlighted. The propeptide residues that are cleaved in the mature protein *in vivo* are shown in grey. The asterisk indicates the fully conserved residues, the colon and period indicate conservation between groups of strongly similar properties scoring> 0.5 and < =  0.5 correspondingly in the Gonnet PAM 250 matrix [49].

Similarly to other microbial rhodopsins, *Hm*BRI is organized as a bundle of seven transmembrane helices (A-G) connected by relatively short loops, with the exception of the elongated beta hairpin-forming B-C loop, which caps the extracellular side, and the extended D-E loop that is unique to *Hm*BRI (Figures 4A and 5). The root mean square deviation (RMSD) of the backbone heavy atoms positions between the *Hm*BRI and *Hs*BR (PDB ID 1C3W) structures is ∼0.6 Å. In the native *Hm*BRI, all the important proton translocation pathway residues, corresponding to D96, D85 and D212, R82, E194 and E204 in *Hs*BR, are conserved. For the crystallization trials, we have used the D94N mutant of *Hm*BRI (which has higher expression level), corresponding to the D96N mutant of *Hs*BR. This mutation is not expected to introduce the structural changes in the protein ground state other than in the vicinity of D94 [2], [30]. Consequently, we will start with the discussion of the retinal binding pocket and the proton release group of *Hm*BRI.

At the core of the *Hm*BRI retinal binding pocket is the cofactor retinal in the all-*trans* conformation (Figure 4B). Bound to the Schiff base is the water molecule W402, coordinated by the aspartates 83 and 216. This arrangement, observed in bacteriorhodopsin-like proton pumps [2], [23] as well as in proteorhodopsins [15], [16] appears to be critical for the proton pumping.

The proton release group configuration in *Hm*BRI is similar to that in the highest-resolution ground state *Hs*BR structure (PDB ID 1C3W), as the R80*_Hm_* side-chain (corresponds to R82*_Hs_*) is turned towards the Schiff base, and the E198*_Hm_* (E194*_Hs_*) and E208*_Hm_* (E204*_Hs_*) side-chains are close to each other (Figures 4C and 4D). Positions of the water molecules, such as 401, 403, 404, 405 and 407, are similar to those in *Hs*BR (Figure 4D). However, the extracellular part of the *Hm*BRI's helix G appears to be slightly unlatched, which results in its outward displacement. As a consequence, there is more water-accessible space in the *Hm*BRI proton release region, and two more water molecules are observed (Figures 4C and 4D). It is not clear how this alteration could affect the proton release mechanism [2], which possibly involves formation of Zundel cation [8], [31].

Finally, we analyze the *Hm*BRI proton donor region and the effects of the introduced *Hm*BRI mutation D94N and the P50*_Hs_* → T48*_Hm_* substitution (Figure 4E). Structures of the D96N and P50A mutants of *Hs*BR have been determined before [30], [32] and allow us to compare the effect of double “mutation” in *Hm*BRI. Structure of the *Hs*BR D96N mutant reveals the outward motion of T46 side-chain, which is hydrogen-bonded to this residue 96. At the same time, positions of the residues close to T46 in sequence, such as P50, remain unchanged (Figure 4E). In the P50A *Hs*BR mutant, on the contrary, the backbone around T46 is relatively unchanged, meanwhile the mutated A50 is moved away from the helix C. The *Hm*BRI D94N structure reveals the most pronounced motion of the helix B relative to the helix C, which seems to be explained by the combination of the effects of the mutation D94N and substitution P50*_Hs_* → T48*_Hm_*. It must be noted that, while the structures of ar-1 and ar-2 are closer to *Hs*BR in this region, dr-3 also has P50*_Hs_* → T substitution (Figure 5). Its structure is intermediate between *Hm*BRI and *Hs*BR P50A mutant [13], [32].

### Novel inter-helical hydrogen bonds in HmBRI

**Figure 6:**
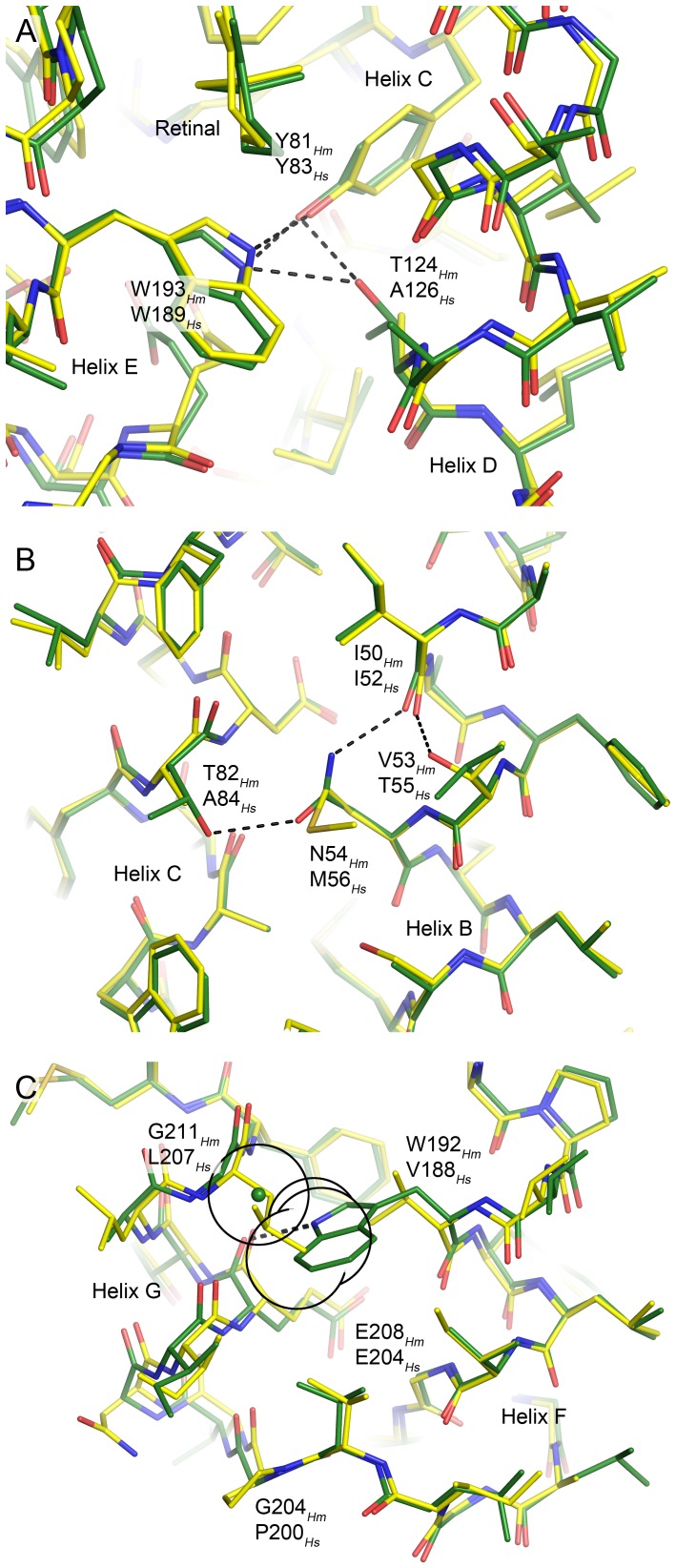
The novel inter-helical hydrogen bonds in *Hm*BRI (green) relative to HsBR (yellow). In each case, the structure alignment was done locally to emphasize the local effects. (A) The substitution A126*_Hs_* → T124*_Hm_* results in two novel hydrogen bonds connecting the helix D to helices C and E. (B) The coupled substitution M56*_Hs_* → N54*_Hm_*, A84*_Hs_* → T82*_Hm_* results in introduction of the hydrogen bond between the helices B and C. Interestingly, the intra-helical hydrogen bond between the I52 backbone oxygen and T55 is replaced with the hydrogen bond between the homologous I50 backbone oxygen and N54 side-chain amine. (C) Coupled substitution V188*_Hs_* → W192*_Hm_*, L207*_Hs_* → G211*_Hm_* results in introduction of the hydrogen bond between the helices F and G (E208 backbone oxygen and W192 indole nitrogen). Interestingly, the glycine is the only possible amino acid at the position 211, as the C_β_ atom of any other amino acid would create a steric conflict with W192 side-chain. The hypothetical position of the residue 211 C_β_ atom is shown by the green sphere, and its van der Waals radius, as well as those of proximal W192 heavy atoms, is shown as a black circle. Introduction of the bulky tryptophan at the position 192 might be another reason for the divergence of the helices F and G in *Hm*BRI, and, as a consequence, the bigger water-accessible volume of the proton release region.

There are several local alterations present in the *Hm*BRI structure relative to the structure of *Hs*BR and other bacteriorhodopsin-like proton pumps resulting from amino acid substitutions. First, T124 of *Hm*BRI helix D is hydrogen-bonded to the tyrosyl oxygen of the helix C tyrosine 81 and to the indole nitrogen of the helix F tryptophan W193 (Figure 6A). While Y81 and W193 that are situated in the vicinity of the retinal β-ionone ring are extremely conserved among archaeal rhodopsins, the threonine amino acid is observed only in *Hm*BRI (Figure 5).

Then, there are two additional inter-helical hydrogen bonds in the hydrophobic regions of *Hm*BRI, which are introduced by coupled substitutions of amino acids.

The first coupled substitution, M56*_Hs_* → N54*_Hm_* and A84*_Hs_* → T82*_Hm_* results in introduction of the hydrogen bond between the helices B and C (Figure 6B). Interestingly, the intra-helical hydrogen bond between the I52 backbone oxygen and T55 side-chain that existed in *Hs*BR is replaced with the hydrogen bond between the homologous I50 backbone oxygen and N54 side-chain amine in *Hm*BRI. None of these bonds is present in ar-1, ar-2 or dr-3.

The second coupled substitution V188*_Hs_* → W192*_Hm_* and L207*_Hs_* → G211*_Hm_* results in introduction of the hydrogen bond between the helices F and G (namely, between the E208 backbone oxygen and W192 indole nitrogen; Figure 6C). Similar tryptophan nitrogen – backbone oxygen hydrogen bonds have already been observed in retinylidene proteins, however, always in special cases. The W138-P186 bond in *Hs*BR (W142-P190 in *Hm*BRI) links the helices E and F, moved away from each other by the intercalating retinal. The similar bond in proteorhodopsins (residues W154-I187 in ESR) brings the cytoplasmic end of the helix F closer to the helix E, but causes a transformation of the α-helix to a complex conformation including a π-helix like element [15]. In *Hm*BRI, glycine is the only possible amino acid at the position 211, as the C_β_ atom of any other amino acid would create a steric conflict with the W192 side-chain (Figure 6C). Thus, *Hm*BRI structure presents an example of the inter-helical hydrogen bond involving tryptophan that does not result in disruption of normal α-helical structure. Interestingly, the distance between the helices F and G is also elevated in ar-1 and ar-2, the reason for which is most probably the bigger-volume leucine amino acid at the place of *Hs*BR's valine. In dr-3, despite an additional aminoacid in the F-G loop, the distance between the helices F and G is the same as in *Hs*BR.

Although the stabilization of membrane proteins due to the hydrogen bonds in the transmembrane region might be moderate [33], presence of three additional bonds at the same time should undoubtedly stabilize *Hm*BRI and might be a reason for its high *E. coli* expression level [20].

### HmBRI trimers and its D-E loop

**Figure 7:**
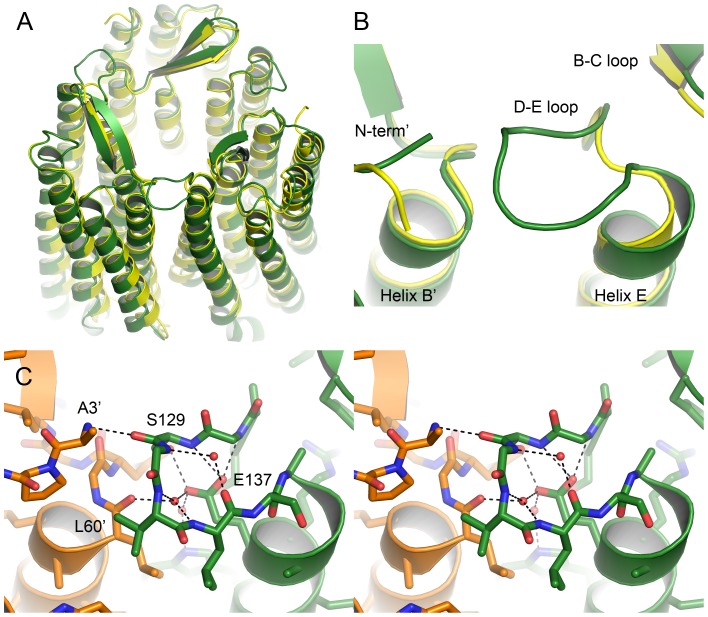
Structure of the *Hm*BRI trimer and its D-E loop. (A) Comparison of the *Hm*BRI trimer structure (green) with that of *Hs*BR [23] (yellow). *Hm*BRI trimer aligns well in the extracellular region, but the protomers are slightly rotated at the cytoplasmic side. (B) Magnification of the D-E loop. Unlike in other trimerizing retinylidene proteins, in *Hm*BRI the loop is extended and makes contact to the adjacent protomer. (C) Wall-eyed stereogram of the *Hm*BRI D-E loop. The adjacent protomer is shown in orange and its residues are marked by a prime. Three structural water molecules are observed that stabilize the loop structure.

Similarly to other archaeal proton pumps [10]–[13] and despite being heterologously expressed in a bacterium *Escherichia coli*, *Hm*BRI assembles as a trimer in crystals (Figure 7A). The RMSD between the backbone heavy atoms positions in the *Hm*BRI and *Hs*BR trimers is ∼1.0 Å. This value reflexes the slightly different orientation of the *Hm*BRI protomers in crystals, where the extracellular sides of the trimers align well, but the cytoplasmic sides are slightly displaced (Figure 7A). Such trimeric organization of *Hm*BRI is similar to that of deltarhodopsin-3 [13], as the RMSD of atomic positions is ∼0.45 Å when either monomers or trimers are compared.

However, there is an important detail that separates *Hm*BRI from the others bacteriorhodopsin-like proton pumps (Figure 7B). The *Hm*BRI's D-E loop is 6 residues longer than the loops of other trimeric proton pumps and makes contacts with the adjacent protomer (Figures 5 and 7B-C). At the core of the loop are the buried E137 side-chain and three structural water molecules, participating in numerous hydrogen bonds (Figure 7C). The loop expands the trimerization interface and might stabilize the trimeric assembly.

### Lipid molecules around HmBRI

**Figure 8:**
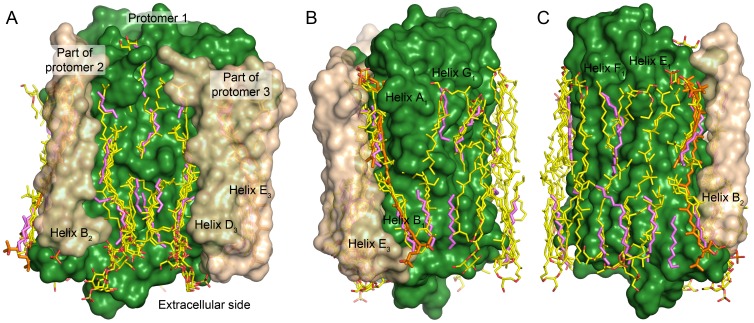
Comparison of the ordered lipidic tails observed in *Hm*BRI structure with those observed in the structures of trimeric *Hs*BR [23], [34]–[36], ar-2 [29] and dr-3 [13]. The *Hm*BRI surface is shown in green, parts of the adjacent protomers are in beige, *Hm*BRI lipids are in magenta, the other lipids are in yellow and bacterioruberin molecules observed in aR-2 and dR-3 structures are in orange. (A) Lipids inside the trimer. (B) Lipids close to the helices A, B and G. (C) Lipids close to the helices E and F. To obtain the positions of the lipidic moieties observed in the structures of the other proteins, their trimeric assemblies were aligned first to *Hm*BRI trimer.

Interestingly, all the retinylidene proteins, for which the trimeric assembly was observed in crystal, were purified from natural or natural-like sources [12], [13], [23], [34]–[38]. The *Hm*BRI crystals, presented here, were grown with the protein expressed in *Escherichia coli*, a bacterium, although the protein itself originates from the archaeon *Haloarcula marismortui*. The ordered lipid tails, belonging either to the *E. coli* lipids or the *in meso* crystallization matrix lipid monooleoyl, are observed at the same positions as the native lipids in other structures (Figure 8). First, there are three paired hydrophobic tails at the extracellular side of the intra-trimer cavity of *Hm*BRI (Figure 8A), where binding of highly specific sulfated triglycoside lipid S-TGA-1 is observed in *Hs*BR crystals [34]–[36]. Second, there are ordered hydrophobic tails on the outer surface of the trimer (Figures 8B and 8C), whose position is also very similar to that in the crystals of *Hs*BR and other proteins. It is remarkable that the lipid binding mode is conserved not only across the different proteins of the family but even across the different kinds of the hydrophobic tail moieties of the lipid molecules (branched isoprenoid chains in archaea and straight fatty acid chains in bacteria). Thus, stronger ability to assemble into a trimer and bind the host lipids might be another reason for the efficient expression of *Hm*BRI in *E. coli* cells.

## Conclusions

Here, we have presented the structure of the D94N mutant of *Haloarcula marismortui* bacteriorhodopsin I at the resolution of 2.5 Å. The protein is trimeric in the crystal. Its retinal-binding pocket and proton acceptor site are similar to those of other archaeal proton pumps, but its proton release region is extended and contains additional water molecules. There are three novel inter-helical hydrogen bonds and a unique extended loop between the helices D and E that makes contacts with the adjacent protomer and might stabilize the trimer. Many lipidic hydrophobic tail groups are discernible in the membrane region, and their position is similar to that of archaeal lipids in the crystals of other proton pumps, isolated from native or native-like sources. Being easily expressed in *E. coli* and crystallized using the *in meso* approach, *Hm*BRI can become a complementary model for studies of the light-driven proton pumping and more general topics such as the link between the protein structure, stability, lipid binding and expression level.

## Materials and Methods

### Construction of Expression Plasmid

*Haloarcula marismortui* bacterio-opsin gene (*bop*, UniProt ID Q5UXY6) with D94N mutation was synthesized *de novo*. The nucleotide sequence was optimized for *E. coli* expression using the GeneOptimizer software (Life Technologies, USA). The gene was introduced into the pSCodon1.2 expression vector (Staby Codon T7, Eurogentec, Belgium) via *Nde*I and *Xho*I restriction sites, resulting in pSC-o*Hm*BRI-His_6_ expression plasmid. Consequently, the expressed construct harbored an additional C-terminal tag with a sequence LEHHHHHH.

### Protein Expression and Purification

An E. coli strain SE1 cells (Staby Codon T7, Eurogentec, Belgium) were transformed with the pSC-o*Hm*BRI-His_6_ plasmid. The cells were grown at 37°C in shaking baffled flasks in an auto-inducing medium ZYP-5052 [39] containing 100 mg/L ampicillin. After the glucose level in the growing bacterial culture dropped below 10 mg/L, the incubation temperature was reduced to 20°C and incubation continued overnight. Collected cells were disrupted using the M-110P Lab Homogenizer (Microfluidics) at 25000 psi in a buffer containing 20 mM Tris-HCl pH 8.0, 5% glycerol and 50 mg/L DNase (Sigma-Aldrich, USA). Membrane fraction of cell lysate was obtained by ultracentrifugation at 90000 g for 1 h at 4°C. The pellets were resuspended in a buffer containing 20 mM Tris-HCl pH 8.0, 0.1 M NaCl and 1% DDM (Anatrace, Affymetrix, USA). All-trans-retinal (Sigma-Aldrich, USA) was added to 10 µM and immediate red shift of the solution color was observed. The mixture was left overnight for solubilization. Insoluble fraction part was removed by ultracentrifugation at 90000 g for 1 h at 4°C. The supernatant was loaded on Ni-NTA column (Qiagen, Germany) and the His-tagged protein was eluted in a buffer containing 20 mM Tris-HCl pH 7.5, 0.1 M NaCl, 50 mM EDTA, 0.01% DDM. The eluate was subjected to size-exclusion chromatography (125 ml Superdex 200 PG, GE Healthcare Life Sciences, USA) in a buffer containing 50 mM NaH_2_PO_4_/Na_2_HPO_4_ pH 7.5, 0.1 M NaCl, 0.01% DDM. Five protein-containing colored fractions were collected, pooled and concentrated to 40 mg/ml for crystallization.

### Spectroscopic characterization

Absorption spectra of *Hm*BRI in solution were collected using the UV-2401PC spectrometer (Shimadzu, Japan). To measure the *Hm*BRI absorption spectra in *H. salinarum* polar lipids, the protein was reconstituted into the liposomes: the polar lipids were added to the solubilized protein in the ratio 3∶1 by mass and the detergent was removed by overnight incubation with Bio-Beads SM-2 (Bio-Rad, USA) at 4°C. Absorption spectra of *Hm*BRI in crystals were collected at the ID-29S cryobench laboratory at the European Synchrotron Radiation Facility (ESRF), Grenoble, France [40] and corrected for the background with a linear function to match the scattering profiles of the protein in solution.

### Crystallization details

The crystals were grown using the *in meso* approach [26], [27], similarly to our previous work [15], [28]. The solubilized protein in the crystallization buffer was added to the monooleoyl-formed lipidic phase (Nu-Chek Prep, USA). The best crystals were obtained using the protein concentration of 20 mg/mL and the 0.1M Tris pH 8.8 and 2.6M Ammonium Sulfate precipitate solution from the Qiagen Cubic Phase I screening kit (Qiagen, Germany). Crystallization trials were set up using the NT8 robotic system (Formulatrix, USA). The crystals were grown at 22°C. Regular shaped hexagonal crystals reaching 100 µm in size appeared in approximately 4 weeks.

### Acquisition and treatment of diffraction data

X-ray diffraction data (wavelength 0.976 Å) were collected at the beamline ID23-1 of the ESRF, using a PILATUS 6M detector. Diffraction patterns were integrated using MOSFLM [41]. The diffraction data were analyzed using the POINTLESS software [42], and the space group was determined to be P321. The reflexes' intensities were scaled using the SCALA software from the CCP4 program suite [43]. The crystals were twinned, with the twin fraction in the range 0–25%. The crystal with the best diffraction was not twinned and was used for structure determination. The data were collected in two wedges from different parts of the same crystal. The diffraction was slightly anisotropic (I/σI of ∼2.5 at 2.5 Å along the directions *a* and *b*, I/σI of ∼2.0 for all the reflections in the range 2.5÷2.65 Å along the direction *c*). The data statistics are presented in the Table 2.

### Structure determination and refinement

Initial phases were successfully obtained in the P321 space group by a molecular replacement (MR) method using MOLREP [44]. There is one monomer in the asymmetric unit in this space group. The *Hm*BRI homology model for MR search was built using the automatic modeling server RaptorX [45]. The R-factors were ∼45% immediately after the MR step and ∼40% after 4 cycles of automatic refinement with REFMAC5 [46]. The initial MR model was then iteratively refined using REFMAC5 [46] and Coot [47]. The final steps of the refinement were conducted using the PHENIX software [48].

### Accession codes

Coordinates and structure factors have been deposited in the Protein Data Bank under the accession code 4PXK.
